# Diurnal Cortisol Patterns and Dexamethasone Suppression Test Responses in Healthy Young Adults Born Preterm at Very Low Birth Weight

**DOI:** 10.1371/journal.pone.0162650

**Published:** 2016-09-12

**Authors:** Nina Kaseva, Riikka Pyhälä, Anu-Katriina Pesonen, Katri Räikkönen, Anna-Liisa Järvenpää, Sture Andersson, Johan G. Eriksson, Petteri Hovi, Eero Kajantie

**Affiliations:** 1 Chronic Disease Prevention Unit, Department of Health, National Institute for Health and Welfare, Helsinki, Finland; 2 Institute of Behavioural Sciences, University of Helsinki, Helsinki, Finland; 3 Folkhälsan Research Centre, Helsinki, Finland; 4 Children’s Hospital, Helsinki University Central Hospital and University of Helsinki, Helsinki, Finland; 5 Department of General Practice and Primary Health Care, University of Helsinki and Helsinki University Hospital, Helsinki, Finland; 6 Vasa Central Hospital, Vasa, Finland; 7 PEDEGO Research Group, Medical Research Center Oulu, Oulu University Hospital and University of Oulu, Oulu, Finland; Centre Hospitalier Universitaire Vaudois, FRANCE

## Abstract

**Background:**

Early life stress, such as painful and stressful procedures during neonatal intensive care after preterm birth, can permanently affect physiological, hormonal and neurobiological systems. This may contribute to altered programming of the hypothalamic-pituitary-adrenal axis (HPAA) and provoke changes in HPAA function with long-term health impacts. Previous studies suggest a lower HPAA response to stress in young adults born preterm compared with controls born at term. We assessed whether these differences in HPAA stress responsiveness are reflected in everyday life HPAA functioning, i.e. in diurnal salivary cortisol patterns, and reactivity to a low-dose dexamethasone suppression test (DST), in unimpaired young adults born preterm at very low birth weight (VLBW; <1500 g).

**Methods:**

The participants were recruited from the Helsinki Study of Very Low Birth Weight Adults cohort study. At mean age 23.3 years (2.1 SD), 49 VLBW and 36 controls born at term participated in the study. For cortisol analyzes, saliva samples were collected on two consecutive days at 0, 15, 30 and 60 min after wake-up, at 12:00 h, 17:00 h and 22:00 h. After the last salivary sample of the first study day the participants were instructed to take a 0.5 mg dexamethasone tablet.

**Results:**

With mixed-effects model no difference was seen in overall diurnal salivary cortisol between VLBW and control groups [13.9% (95% CI: -11.6, 47.0), *P* = 0.31]. Salivary cortisol increased similarly after awakening in both VLBW and control participants [mean difference -2.9% (29.2, 33.0), *P* = 0.85]. Also reactivity to the low-dose DST (awakening cortisol ratio day2/day1) was similar between VLBW and control groups [-1.1% (-53.5, 103.8), *P* = 0.97)].

**Conclusions:**

Diurnal cortisol patterns and reactivity to a low-dose DST in young adulthood were not associated with preterm birth.

## Introduction

Cortisol secretion shows a diurnal pattern with a decrease from morning to evening levels [1]. This diurnal rhythm is an important marker of hypothalamic-pituitary-adrenal axis (HPAA) regulation [2], and it is established during the first year of life [3], in some infants even at 2 months of age [4]. It has been suggested that pre- and postnatal stress, and subsequent cortisol exposure, could alter and “program” HPAA function and thus affect health over the life course [5]. In infants born preterm, frequent invasive, painful and stressful treatment procedures performed during neonatal intensive care, are examples of such early life stress. Furthermore, the preterm infant with immature physiological systems, is particularly vulnerable during this time of early life stress and simultaneous programming of the stress systems [6].

Increased cortisol exposure, normally tightly regulated by the HPAA, is associated with development of several adverse health outcomes, including cardiovascular disease, osteoporosis and insulin resistance [7]. Similarly, preterm birth is linked with an increased risk for these conditions. As adults, individuals born preterm at very low birth weight (VLBW, ≤ 1500 g), have higher blood pressure [8,9], changes in lipid profile [9,10], lower mineral bone density [11,12] and impaired glucose regulation [12,13], compared with peers born at term. Based on current literature, HPAA functioning, especially in relation to stress responses, may be altered in those born preterm [14–20], although the results have been inconsistent. Assuming that preterm birth provokes durable changes in adult HPAA function, this could modify adult health. To clarify the role of preterm birth on adult HPAA function we examined diurnal cortisol patterns and cortisol feedback inhibition by a low-dose dexamethasone suppression test (DST) in healthy young adults born preterm at VLBW and controls born at term.

## Methods

### Participants

**Fig 1:**
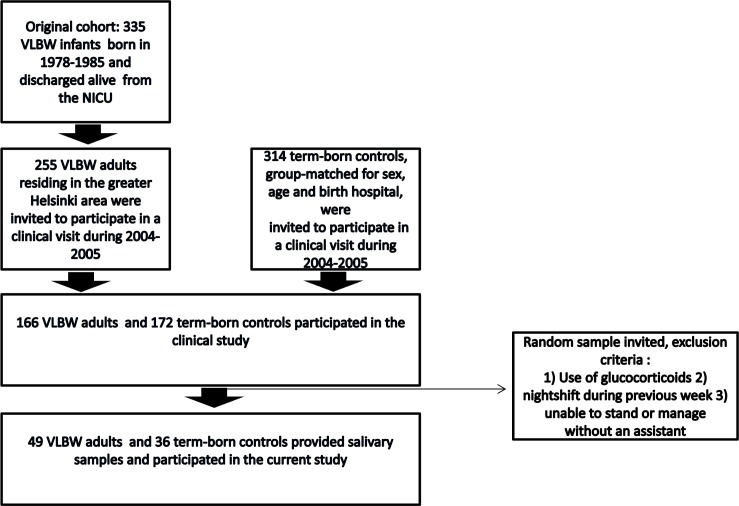
Flow chart of the study population.

The data come from the Helsinki Study of Very Low Birth Weight Adults, a case-control cohort which has previously been described in detail [13]. The original cohort included 335 subjects born preterm at VLBW during 1978–1985 and discharged alive from the neonatal intensive care unit serving one regional area in southern Finland, and 314 controls born at term, group-matched for sex, age and birth hospital. In 2004–2005, those 255 VLBW and 314 control subjects who were residing in the greater Helsinki area were invited to participate in a clinical examination. Of these invited subjects, 166 VLBW and 172 controls chose to participate. During 2005 a random sample among the participants (166 VLBW and 172 controls) of the cohort’s first clinical visit in 2004–2005 was invited to participate in the current study, selected as previously described [20]. Briefly, we excluded individuals who 1) used glucocorticoids, 2) had a nightshift during the previous week or 3) were unable to stand or manage without an assistant. A total of 49 VLBW (26 men and 23 women) and 36 controls (14 men and 22 women) provided adequate salivary samples and were included in the analyses (Fig 1). Anthropometry was measured and all participants completed a detailed questionnaire regarding health status, including smoking habits, medical history and medications.

#### Ethics

The study was performed according to the Declaration of Helsinki. The Ethics Committee of the Helsinki and Uusimaa Hospital District approved the study protocol. Written informed consent was obtained from each participant.

#### Non-participant analysis

We compared perinatal and current descriptive characteristics between the participants of this study (49 VLBW and 36 controls) and the group they were recruited from (Fig 1), i.e. the remaining cohort that previously participated in a clinical examination (117 VLBW and 136 controls) (13). The sample was comparable to the birth cohort in terms of gestational age, birth weight, sex, incidence of pre-eclampsia and multiple pregnancy (all *P*-values ≥ 0.1). Of the sample, 47% of VLBW participants were small for gestational age (SGA) [21] compared to 28% in the group they were recruited from (*P* = 0.03). Age, daily smoking, hormonal contraception use, parental education, adult height and body mass index (BMI) were also similar between the participants of the current study and the group they were recruited from (all *P*-values ≥ 0.1).

### Measures and procedures

As a marker of HPAA function we used diurnal salivary cortisol profiles. We collected saliva samples using Salivette® (Sarstedt, Nümbrecht, Germany) on two consecutive days at 0, 15, 30 and 60 min after wake-up, at 12:00 h, 17:00 h and 22:00 h.

After the last salivary sample of the first study day, at 22:00 h, the participants were instructed to take a 0.5 mg dexamethasone tablet. The cortisol response to the DST was calculated as the difference between log-transformed awakening cortisol on days 2 and 1, back-transformed to indicate the Day 2/ Day 1 ratio. We chose this dose of dexamethasone because our aim was to assess individual variation in normal HPAA suppression rather than to assess endocrine disorders, for which higher doses are used in routine endocrine practice.

The cortisol concentrations were determined using a competitive solid-phase, time-resolved fluorescence immunoassay with fluorometric end point detection (DELFIA, Wallac, Turku, Finland). Cortisol measurements were assayed in duplicate (University of Trier, Germany).

#### Statistical analyses

All statistical analyses were conducted with IBM SPSS Statistics 21 (SPSS Inc., Chicago, IL, USA). The significance level was set to two-tailed *P* < 0.05. We compared descriptive characteristics between VLBW and control groups with *t*-test (continuous variables) and χ^2^-test (categorical variables).

To attain normality, cortisol concentrations were log-transformed. We analyzed overall diurnal cortisol with mixed-effects models, which has the advantage of taking into account within-subject correlation in studies with repeated measurements. We adjusted for age and sex in model 1; for age, sex and childhood socioeconomic status (SES; highest parental education) and maternal smoking during pregnancy in model 2; and for age, sex, highest parental education, maternal smoking during pregnancy, BMI, smoking and use of hormonal contraception in model 3. We additionally adjusted for menstrual cycle phase in model 4 if data was available. The results are presented as mean differences (%) and 95% confidence intervals (CI) between VLBW and control groups.

We tested if VLBW and control groups have different trajectories of diurnal cortisol by including an interaction term with sampling time (time*VLBW status). We also assessed sex differences by interaction terms (sex*VLBW status and sex*time*VLBW status).

To compare common indicators of diurnal cortisol patterns, i.e. awakening, peak after awakening, cortisol awakening response (CAR) and 22:00 h concentrations, we used multiple linear regression models. CAR describes the increase in cortisol concentration 30–45 min after awakening and reflects the adrenal capacity to stress response [22]. The CAR was calculated as 1) peak after awakening minus value at awakening and 2) as the area under the curve (AUC), calculated from the first four salivary samples (0, 15, 30 and 60 min post awakening). Diurnal AUC was calculated from all seven salivary samples collected (0, 15, 30 and 60 min post awakening and 12:00, 17:00 and 22:00 h).

## Results

Within the VLBW group, gestational age ranged between 24.1 and 35.6 weeks (mean 29.4 weeks), and within controls between 38.0 and 42.3 weeks (mean 40.2 weeks). Birth weights ranged between 600 and 1500 g (mean 1099 g) and between 2880 and 4900 g (mean 3641 g), respectively. Detailed descriptive characteristics of the participants are shown in Table 1.

**Table 1:** Descriptive characteristics of the participants.

Characteristic	VLBW (n = 49)	Term (n = 36)	*P* ^a^
**Birth**			
Gestational age, mean (SD), week	29.3 (2.4)	40.2 (1.1)	<0.0001
Birth weight, mean (SD), g	1099 (202)	3641 (473)	<0.0001
Birth weight SDS, mean (SD)	-1.6 (1.5)	0.2 (1.1)	<0.0001
Women, n (%)	23 (46.9)	22 (61.1)	0.5
Men, n (%)	26 (53.1)	14 (38.9)	0.4
SGA ^b^, n (%)	23 (46.9)	0	
Preeclampsia, n (%)	9 (20.0)	5 (14.3)	0.5
Twin, n (%)	6 (12.2)	0	
Triplet, n (%)	1 (2.0)	0	
**Current**			
Age, mean (SD), y	23.3 (2.1)	23.6(2.3)	0.6
Height, mean (SD), cm			
Women	161.5 (5.8)	166.2 (6.9)	0.02
Men	174.4 (8.0)	181.1 (4.8)	0.007
Body mass index, mean (SD), kg/m^2^			
Women	21.7 (3.2)	23.9 (4.8)	0.07
Men	22.5 (4.1)	23.4 (3.3)	0.5
Daily smoking, n (%)	14 (28.6)	10 (28.6)	1.0
Menstrual cycle phase ^c^ if no hormonal contraception, n (%)			0.3
Day 1–8	8	10	
Day 9- end of cycle	9	7	
History of depression diagnosed by a physician, n (%)	6 (12.2)	5 (13.9)	0.8
Use of hormonal contraceptives, n (%)	12 (52.2)	7 (31.8)	0.2
Parental education, n (%)			0.07
Elementary	4 (8.3)	2 (5.7)	
High school	14 (29.2)	3 (8.6)	
Intermediate	18 (37.5)	14 (40.0)	
University	12 (25.0)	16 (45.7)	

VLBW, very low birth weight (<1500 g)

^a^T-test for continuous and chi-square test for categorical variables

^b^ SGA, small for gestational age, birth weight < -2 SD

^c^ Data available for 17 VLBW and 17 control women

### Cortisol awakening response (CAR)

As expected, salivary cortisol increased after awakening in both VLBW and control groups (Table 2). We found no differences in CAR between VLBW and control groups [-2.9% (95% CI: 29.2, 33.0), *P* = 0.85].

**Table 2:** Diurnal salivary cortisol of the study participants, before (Day 1) and after (Day 2) a low-dose dexamethasone suppression test.

	VLBW (n = 49) Mean ^a^ (SD ^b^)	Term (n = 36) Mean ^a^ (SD ^b^)	Mean difference ^c^ (95% CI)	*P* ^d^
**Diurnal salivary cortisol (nmol/l) day 1**				
Upon awakening	7.6 (2.2)	7.8 (1.7)	-1.4 (-28.9, 37.1)	.94
Peak after awakening	15.3 (1.7)	15.8 (1.4)	-2.9 (-22.4, 21.1)	.78
Awakening response ^e^	2.0 (1.9)	2.0 (1.9)	-2.9 (29.2, 33.0)	.85
Awakening AUC ^f^	10.7 (1.7)	11.4 (1.4)	-4.3 (22.9, 18.9)	.68
Diurnal AUC ^g^	4.0 (2.2)	3.4 (1.6)	18.9 (-14.7, 66.0)	.30
At 22:00 hours, before taking dexamethasone	1.5 (4.0)	1.0 (2.2)	55.6 (-12.1, 74.8)	.13
**Diurnal salivary cortisol (nmol/l) day 2**				
Upon awakening	0.6 (5.6)	0.4 (3.2)	24.5 (-39.5, 55.9)	.55
Peak after awakening	1.0 (5.3)	1.1 (3.2)	-13.7 (-56.2, 69.8)	.67
Awakening response ^e^	1.4 (2.4)	2.3 (2.4)	-35.3 (-57.4, -1.8)	**.04**
Awakening AUC ^f^	0.6 (5.0)	0.6 (3.4)	-13.3 (-56.5, 73.0)	.68
Diurnal AUC ^g^	0.6 (4.7)	0.6 (3.2)	-6.7 (-52.8, 84.1)	.84
At 22:00 hours	0.6 (7.6)	0.5 (3.9)	-1.8 (-55.9, 18.8)	.96
Dexamethasone suppression response ^h^	0.08 (6.5)	0.06 (3.9)	-1.1 (-53.5, 103.8)	.97
Ratio of awakening AUC day 2/day 1^i^	0.06 (3.6)	0.06 (3.2)	-5.6 (-46.7, 67.5)	.85
Ratio of diurnal AUC day 2/Diurnal AUC day 1 ^j^	0.15 (2.7)	0.17 (3.1)	-20.4 (-51.4, 30.3)	.36

VLBW, very low birth weight (<1500 g)

^a^ Geometric mean, denotes the nth root of the product of n individual values

^b^ Geometric standard deviation, denotes the relative increase in a variable corresponding to one standard deviation unit change in the logarithm of the variable

^c^ Linear regression model 1, adjusted for age and sex

^d^ *P* for linear regression model 1, adjusted for age and sex

^e^ Peak value after awakening minus value at awakening

^f^ Area under the curve (AUC) ground (above zero), calculated from the first four salivary samples, collected 0, 15, 30 and 60 min after awakening

^g^ AUC ground (above zero), calculated from salivary samples collected 0, 15, 30 and 60 min after awakening and at 12:00, 17:00 and 22:00 hours

^h^ Cortisol upon awakening on day 2 minus day 1, reflects response to the dexamethasone test

^i^ Awakening AUC day 2 minus day 1, back-transformed from log-transformed values, gives the ratio of salivary cortisol after the dexamethasone test

^j^ Diurnal AUC day 2 minus day 1 back-transformed from log-transformed values, gives the ratio of salivary cortisol after the dexamethasone test

### Diurnal cortisol

**Fig 2:**
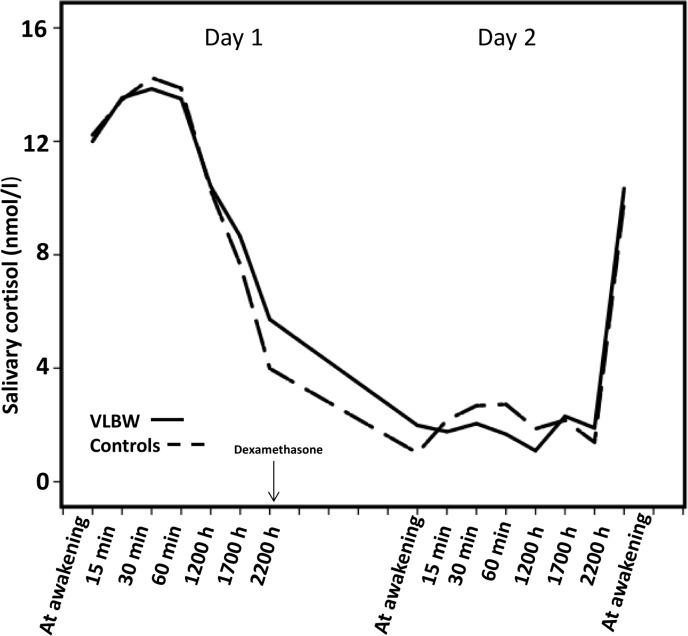
Diurnal cortisol patterns and dexamethasone suppression test responses of the participants. Diurnal salivary cortisol concentrations during Day 1 and after a low-dose dexamethasone suppression test (Day 2) among adults born preterm at very low birth weight (VLBW; <1500g) (continuous lines) and controls born at term (dashed lines).

Diurnal cortisol patterns were similar in VLBW and control groups (Fig 2). With mixed model no difference was seen in overall diurnal cortisol [13.9% (95% CI: -11.6, 47.0), *P* 0.31] when adjusting for age and sex. Further adjusting for childhood socioeconomic status in model 2; BMI, smoking and use of hormonal contraception in model 3 and for menstrual cycle phase in model 4 did not change the results (Table 3, all *P*-values ≥ 0.31). Also diurnal AUC and evening cortisol were similar in both groups (Table 2).

**Table 3:** Mixed model results, showing overall differences in daily salivary cortisol concentrations between very low birth weight (VLBW, <1500g) and control participants, presented as mean differences (95% CI).

	Day 1, Mean difference (95% CI)	*P*	Day 2, Mean difference (95% CI)	*P*
**VLBW vs controls, men and women**				
Model 1	13.9% (-11.6, 47.0)	.31	-2.2% (-45.9, 76.9)	.94
Model 2	6.7% (-15.2, 34,3)	.57	-17.1% (-51.9, 42.8)	.50
Model 3	6.5% (-16.6, 36.0)	.61	-14.8% (-51.5, 49.6)	.57
Model 4	15.2% (-23.5, 73.6)	.49	9.7% (-58.8, 192.0)	.85
**VLBW vs controls, men only**				
Model 1	8.8% (-31.3, 72.2)	.71	-16.7% (-68.3, 118.6)	.70
Model 2	-5.3% (-34.9, 37.7)	.77	-39.6% (-72.2, 30.9)	.19
Model 3	-8.7% (-40.0, 39.0)	.66	-34.8% (-71.9, 51.1)	.31
Model 4	NA	NA	NA	NA
**VLBW vs controls, women only**				
Model 1	18.8% (-11.8, 60.0)	.25	9.7% (-49.9, 139.9)	.81
Model 2	17.3% (-13.6, 59.2)	.30	5.5% (-52.4, 133.8)	.89
Model 3	19.9% (-15.3, 67.2)	.31	21.1% (-48.0, 182.0)	.65
Model 4	15.2% (-23.5, 73.6)	.49	9.7% (-58.8, 192.1)	.85
**VLBW-SGA**^**a**^ **vs. VLBW-AGA**^**b**^				
Model 1	39.4% (-11.0, 118.4)	.14	102.5% (-19.6, 410.0)	.13
Model 2	29.3% (-14.3, 195.1)	.21	68.5% (-28.4, 296.2)	.23
Model 3	23.9% (-17.7, 83.6)	.30	50.8% (-33.8, 243.7)	.32
Model 4	12.5% (-56.8, 193.1)	.78	-18.1% (-90.7, 621.8)	.84

VLBW, very low birth weight (<1500 g)

Model 1 adjusted for age and sex

Model 2 adjusted for age, sex, highest parental education and maternal smoking during pregnancy

Model 3 adjusted for age, sex, highest parental education, maternal smoking during pregnancy, BMI, smoking of the participant and use of hormonal contraception

Model 4 adjusted for age, sex, highest parental education, maternal smoking during pregnancy, BMI, smoking of the participant, use of hormonal contraception and menstrual cycle phase if data available

^a^ Small for gestational age (birth weight < -2SD)

^b^ Appropriate for gestational age (birth weight ≥ -2SD)

### Dexamethasone suppression test

After the low-dose DST we found a lower CAR in the VLBW group (1.4 vs. 2.3, *P* = 0.04, Table 2), this difference did not remain statistically significant after further adjusting for confounders in models 2–4. Also the awakening and diurnal AUC were similar in both groups (*P* ≥ 0.68).

### Men and women

We tested for sex, group and time interactions. No two-way or three-way interactions were found in analyses of any outcome measures (mixed model *P*-values for interaction terms across the different adjustment models were as follows: sex*VLBW status ≥ 0.67; time*VLBW status ≥ 0.33; sex*time*VLBW status ≥ 0.57) and therefore the results are presented with men and women combined. As sex differences are a common characteristic of HPAA stress response studies, we reran all analyses with men and women separately (Table 3). Overall diurnal cortisol, comparing men only [8.8% (95% CI: -31.3, 72.2), *P* = 0.71], and women only [18.8% (95% CI:-11.8, 60.0) *P* = 0.25], showed no significant differences between VLBW and control groups.

### History of depression

Of the participants, 6 (12.2%) VLBW and 5 (13.9%) controls had history of depression diagnosed by a physician. We reran all the analyses after excluding the VLBW and control participants with diagnosed depression. The results remained similar.

### Small and appropriate for gestational age

Within the VLBW group 23 (46.9%) participants were born SGA. We reran all the analyses comparing those 23 VLBW born SGA with the 26 born appropriate for gestational age (AGA, birth weight ≥ -2SD). There were no significant differences in diurnal cortisol or DST responses between SGA and AGA VLBW participants (Table 3).

## Discussion

We found no difference in diurnal salivary cortisol patterns in healthy VLBW and control young adults. The results remained after adjustment for important confounders, including age, sex, SES, BMI, smoking, use of hormonal contraception and menstrual cycle phase, as applicable. We further found no difference in the response to a low-dose DST between VLBW and control groups, measured as overall cortisol and AUC (awakening and diurnal). The lower awakening cortisol after the low-dose DST attained nominal statistical significance, but this finding was attenuated after adjustment for confounders.

Neonatal exposure to stress or maternal cortisol can permanently modify HPAA function, and thereby have long-term health impacts [23,24]. Our finding of similar diurnal cortisol patterns in VLBW and control groups suggest similar HPAA function in everyday situations. This is an important finding, taking into account the higher risk of disease and increased risk factors largely reported in VLBW adults [8,9,12,13]. Although prior studies on the effects of preterm birth on diurnal cortisol patterns have been few, one study recently reported lower waking cortisol (P<0.039) and attenuated diurnal cortisol decline from waking to bedtime (P<0.035) in prematurely born men, aged 21–23 years, compared with term-born men [25]. In line with our findings, Lee et al. also reported similar waking cortisol and diurnal cortisol patterns in prematurely born women compared with term-born controls [25]. In contrast to these findings, in an explorative examination, another study showed that individuals born preterm or with small birth weight (< 2500g) had higher evening and total cortisol levels at 43 years of age [26]. We are unaware of any further similar studies in adults. However, Grunau et al. measured hair cortisol levels, an integrated index of endogenous HPAA activity, in preterm children at seven years of age, and reported lower hair cortisol levels in preterm compared to term-born children [18]. This finding suggests persistent alteration of stress system programming in preterm children extending into school age.

Interindividual variations in cortisol concentrations are considerable. We previously reported 17.2% lower plasma cortisol responses [(95% CI: -28.9, -3.5) *P* = 0.02] after a standardized stressor, the Trier Social Stress Test [27], in the same VLBW adults we now report on, when compared with controls born at term [20]. The 95% CI’s of this finding of lower cortisol responses after stress fit in the 95% CI’s of the current results, and we are unable to detect such small differences in this study.

There are limitations to our study. Notably, the sample size is relatively small. This may diminish possibilities of finding associations in subgroups, i.e. evaluation of sexes separately. Furthermore, a higher proportion of the VLBW participants of the current study were born SGA, 47% compared to 28%, than in the group they were recruited from. Lifetime stressful experiences and current perceived stress affect HPAA function and we were unable to control for these factors in the analyses.

Strengths of our study include collecting seven cortisol samples in duplicate on two consecutive days. We were able to adjust for important confounders in the analyses, including use of hormonal contraception. A strength of our study population is the age-, sex- and birth hospital-matched control group.

## Conclusions

Our study contribute to the growing literature on the effects of early life stress on later HPAA functioning. To conclude, although previous literature suggests that HPAA stress response is altered in VLBW individuals [14–20], diurnal cortisol patterns in everyday situations and after a low-dose DST challenge seem similar in healthy VLBW and control young adults.
